# Common orthopaedic trauma may explain 31,000-year-old remains

**DOI:** 10.1038/s41586-023-05756-8

**Published:** 2023-03-15

**Authors:** Nicholas J. Murphy, Joshua S. Davis, Seth M. Tarrant, Zsolt J. Balogh

**Affiliations:** 1 Department of Traumatology, John Hunter Hospital & University of Newcastle, Newcastle, New South Wales Australia; 2 School of Medicine and Public Health, University of Newcastle, Newcastle, New South Wales Australia; 3 Department of Infectious Diseases, John Hunter Hospital, Newcastle, New South Wales Australia

**Keywords:** Medical research, Pathogenesis, Archaeology

arising from T. R. Maloney et al. *Nature* 10.1038/s41586-022-05160-8 (2022)

The fascinating discovery of skeletal remains in Borneo of an individual (TB1) with absent left distal tibia, fibula and foot from 31,000 years ago^1^ has been proposed as evidence of a contemporaneous sophisticated amputation procedure. Maloney et al.^1^ infer from the bony abnormalities that surgical amputation is the only possible explanation and, furthermore, that the limb shows no evidence of infection. We dispute the conclusion that these skeletal remains provide evidence of a transosseous surgical amputation and that the limb shows no signs of infection. We propose that the skeletal findings have more plausible alternative explanations, such as the natural history of an injury pattern commonly encountered in blunt orthopaedic trauma, an open distal tibia/fibula fracture with growth-plate involvement.

From the perspective of orthopaedic trauma surgeons practicing in this field, the exclusion of blunt trauma as a potential mechanism of injury is a rather reductionist approach to a differential diagnostic puzzle with several missing pieces from thousands of years ago. Maloney et al.^1^ dismissed blunt trauma with the assertion that it “typically causes comminuted and crushing fractures”. To support this statement, they cited an isolated case report of an axis fracture^2^ (the second cervical vertebra in the neck). We disagree with the statement and suggest the supporting citation is inadequate. Oblique fracture patterns such as TB1’s are frequently observed from blunt trauma in clinical practice^3^.

Physeal (growth plate) fractures of the distal tibia and fibula are common injuries in adolescents^4^. The most common subtype^5^ involves fracture through the distal tibial physis and into the metaphysis (Salter–Harris type II^6^), and typically occurs concomitantly with a distal fibula fracture^7^. It is not uncommon that the medial apex of the fracture pierces through the skin^8,9^, while the foot displaces laterally, as seen in Fig. 1. The mean age of people presenting with this Salter–Harris type II injury to the distal tibia and fibula is 12–13 years^7^, which corresponds to the predicted age of injury for the individual of whom the skeletal remains were discovered in Borneo, given he or she is estimated to have died aged 19–20 (a predicted 6–9 years after suffering the injury)^1^. The typical mechanism of these injuries is forced inversion or eversion while the foot is fixed in position on the ground. In Borneo 31,000 years ago, this may have eventuated from any slip or misstep whilst running, or a jump from low height. Today, open ankle fractures are managed with intravenous antibiotics, tetanus prophylaxis and expeditious debridement with open reduction and internal fixation in the operating theatre.

**Fig. 1:**
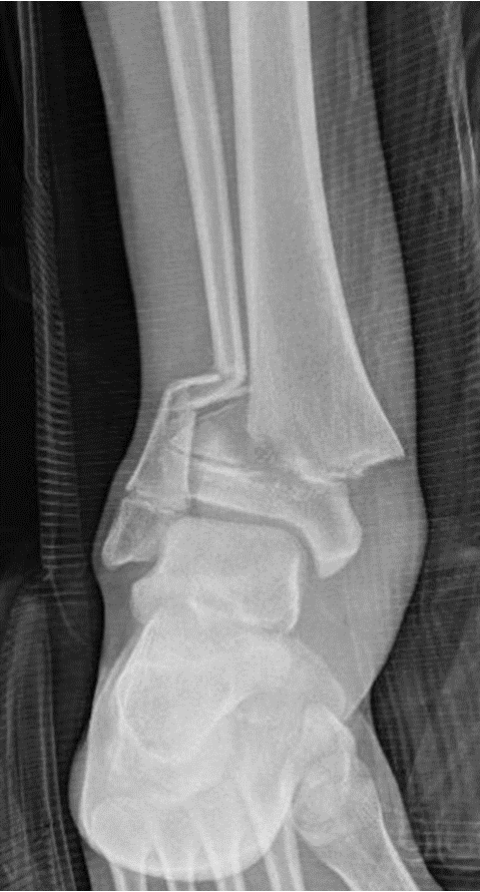
Plain radiograph of a distal tibia and fibula fracture. A plain radiograph of a distal tibia and fibula fracture involving the physis (growth plate) in a 14-year-old individual.

In Borneo 31,000 years ago, the natural history of an open physeal ankle fracture without modern surgical care could quite plausibly have produced the findings encountered in TB1’s skeletal remains. In some cases, the inoculation of bacteria into exposed bone could result in acute infection progressing to overwhelming sepsis and death. In other cases, a chronic osteomyelitis can develop, often with the formation of a life-long draining sinus^10^. Survival with chronic osteomyelitis was described on many occasions in the pre-antibiotic era^11^. In the 1830s, Nathan Smith, a professor of surgery at Yale University, suggested that most people with osteomyelitis he observed survived with the condition, writing “a very great majority of patients survive the attack, albeit with long confinement, protracted suffering and great emaciation.”^12^. The “long confinement, protracted suffering” was most probably TB1’s fate during his young adulthood. It is highly implausible that either surgical amputation or an open fracture, in the absence of antiseptics, anaesthetics or antibiotics, could occur without a subsequent established infection. Furthermore, the bony changes shown in figure 3 of Maloney et al.^1^ are typical of chronic osteomyelitis—the cortical thickening of the distal tibia and fibula are consistent with involucrum, and the small bony defect of the distal tibia could represent an area of sequestrum. The evidence of bone lysis and necrosis at the distal tibial and fibula, which Maloney et al.^1^ refer to in the caption of figure 3b, could quite plausibly occur secondary to infection^13^. The suggested mechanism explains other characteristics of the skeletal findings—the missing malleolar parts of the distal tibia and fibula are consistent with common adolescent fracture patterns, and the small size of the left tibia and fibula relative to the right is highly suggestive of physeal arrest, which is a common complication of displaced physeal fractures^7,14^.

With a distal tibia and fibula fracture already present and necrosis of the bone and surrounding soft tissues occurring due to infection, terminalization of the limb—that is, cutting through the remaining soft tissues—is a more plausible scenario. This is a substantially different proposition from the primary transosseous surgical amputation described in Maloney et al.^1^, which states that the bone must have been cut with a sharp instrument. It is impossible to know whether loss of the foot occurred around the time of injury, or weeks to months later. If an arterial injury accompanied the initial bony injury, and the limb suffered distal ischaemia as a result, a dry gangrenous process may even have autoamputated the limb without any assistance.

We cannot exclude the possibility of rarer causes to explain these skeletal remains—for example, congenital transverse deficiency of the lower limb, a rare congenital anomaly that can manifest as a hypoplastic limb with absent foot^15^. If TB1 were born with this condition, weightbearing on the footless lower limb without a durable heel pad could have caused ulceration and the chronic infective changes that we observe in the bony architecture of TB1’s distal tibia and fibula.

Overall, we find that the conclusions drawn by Maloney et al.^1^ are unconvincing. Performing primary supra-articular transosseous surgical amputation through the thick cortices of the tibia without specialized metallic tools (at least chisel and saw) would be very difficult and is highly improbable. If the people in Borneo were performing lower-limb amputation using ‘sharp instruments’, it would have been easier to perform transarticular amputation through the soft tissues of the ankle joint, where it is not necessary to transect thick cortical bone. This is not the pattern observed in these skeletal remains.

We suggest that interdisciplinary input from expert orthopaedic trauma surgeons and bone and joint infection experts would be of value in archaeological studies such as this to aid in formulating plausible explanations of injury mechanism and infectious processes, as palaeopathology is unlikely to cover the breadth of specialized understanding required. Unfortunately, our concerns are not limited to the explanation of the missing part of the skeleton. Figure 3a of Maloney et al.^1^ contains a photographed reconstruction of TB1’s bony anatomy, with the distal portion of the right tibia placed back-to-front such that the medial malleolus is incorrectly articulating with the distal fibula. As an image that is likely to be frequently reproduced, and that has already had considerable media attention, this requires correction. Although we cannot support the conclusions of Maloney et al.^1^, we nevertheless consider the findings described to be of great interest. Even if TB1 did not undergo a transosseous surgical amputation, the findings demonstrate evidence of an individual who, 31,000 years ago, must have had enormous kin support to survive for several years after a severe open injury, which of itself seems a notable detail about our ancestors.

## Reporting summary

Further information on experimental design is available in the Nature Portfolio Reporting Summary linked to this Article.

## Online content

Any methods, additional references, Nature Portfolio reporting summaries, source data, extended data, supplementary information, acknowledgements, peer review information; details of author contributions and competing interests; and statements of data and code availability are available at 10.1038/s41586-023-05756-8.

## Supplementary information

Reporting Summary

## Data Availability

All relevant data are included in the Article.
